# Reproducibility of Immunohistochemical Testing of Estrogen Receptors, Progesterone Receptors, Human Epidermal Growth Factor Receptor-2 (HER2) and Ki-67 in Vietnam

**DOI:** 10.3389/bjbs.2025.15455

**Published:** 2025-11-05

**Authors:** Thai Anh Tu, Nguyen Van Tin, Anthony Rhodes, Dinh Bui Quynh Anh, Le Thi Hong Dao, Nguyen Thi Truc Linh, Dinh Thi Khanh Nhu, Nguyen Thi Hong Nhung, Lam Thanh Cam, Ngo Thi Minh Hanh, Pham Nguyen Cuong, Nguyen Thanh Toan, Nguyen Khac Tuyen, Do Dinh Khanh, Tran Thi Truc Ngan, Lam Kieu Mong Thy, Nguyen Van Thanh, Nguyen Quang Tuan, Vo Ngoc Nguyen, Le Thi Thuy Nhu, Nguyen Dam Chau Bao

**Affiliations:** 1 Department of Pathology, Ho Chi Minh City (HCMC) Oncology Hospital, Ho Chi Minh City, Vietnam; 2 Bureau of Accreditation Vietnam, Center for Standardization and Quality Control in Medical Laboratory of Ho Chi Minh City (CSQL), Ho Chi Minh City, Vietnam; 3 Institute of Biomedical Sciences, London, United Kingdom; 4 Department of Pathology, Ca Mau General Hospital, Ca Mau, Vietnam; 5 Department of Pathology, 108 Military Central Hospital, Ha Noi, Vietnam; 6 Department of Pathology, Hue Central Hospital, Hue, Vietnam; 7 Department of Pathology, Military Hospital 175, Ho Chi Minh City, Vietnam; 8 Department of Pathology, Nhan dan Gia Dinh Hospital, Ho Chi Minh City, Vietnam; 9 Department of Pathology, Can Tho Oncology Hospital, Can Tho, Vietnam; 10 Department of Pathology, Tu Du Hospital, Ho Chi Minh City, Vietnam; 11 Department of Pathology, Da Nang Oncology Hospital, Da Nang, Vietnam; 12 Department of Pathology, Da Nang Hospital for Women and Children, Da Nang, Vietnam

**Keywords:** ER, PR, HER2, Ki67, breast cancer

## Abstract

**Context:**

Immunohistochemical (IHC) testing of estrogen receptors (ER), progesterone receptors (PR), HER2 and Ki-67 on breast cancer samples is carried out in the majority of clinical departments to predict response to therapies and to determine prognosis. Issues surrounding the reproducibility of testing are well documented and guidelines recommend laboratories participate in external quality assessment (EQA) in order to ensure reliability of results.

**Objective:**

To assess the reproducibility of IHC testing for these markers in hospitals from the south, north, and centre of Vietnam, estimated to be approximately half of all clinical hospitals in the country performing these tests.

**Design:**

As cases are referred for testing between hospitals, an EQA ring study was designed that included the testing of samples from all participating laboratories. Participants were provided with unstained slides of invasive breast carcinomas with different expression levels for ER, PR, HER2 and Ki-67.

**Results:**

There was a significant level of reproducibility for all four biomarkers, with ER testing giving the least variation in results (kappa 0.822, coefficient of variation [CV] 4.8%) and Ki-67 the greatest variation (kappa 0.647 CV 17%). However, 328/392 (84%) and 317/392 (81%) of the Ki-67 evaluations were in agreement when employing the clinically relevant cut points of ≥30% and ≥20%, respectively. The reproducibility of testing for HER2-low expression was relatively poor (kappa 0.323, 95% CI 0.223–0.424), compared to overall agreement for HER2 testing (kappa 0.794, 95% CI 0.753–0.836).

**Conclusion:**

This is the first EQA ring study held within Vietnam for ER, PR, HER2 and Ki-67 and sets the base line as to the current level of reproducibility in the country. Continued participation in the program will help ensure the reliability of testing for clinical use.

## Introduction

Immunohistochemical (IHC) testing for estrogen receptors (ER), progesterone receptors (PR) and human epidermal growth factor receptor −2 (HER-2) is now carried out on the tissue samples of all newly diagnosed patients with invasive breast cancer in the vast majority of hospital laboratories [1, 2]. The results indicate the likely benefit to patients of hormonal therapies (tamoxifen, aromatase inhibitors) targeting estrogen driven tumors or antibody based therapies that target the HER2 oncogene (trastuzumab and trastuzumab deruxtecan) [3–5]. Testing with these markers, in addition to the cell proliferation marker Ki-67, also provides valuable prognostic information by predicting how aggressive a tumor is and consequently if a patient is likely to benefit from chemotherapy [6, 7].

The results of these IHC assays all require some degree of quantitative assessment in order to determine whether or not the tumors express sufficient amounts of receptor to indicate a beneficial response to therapies. However, issues surrounding the reproducibility of IHC testing for ER, PR, HER2 and Ki-67 and their assessment are well documented, to include the negative impact of laboratories producing erroneous results [8–14]. Consequently, to ensure the reliability of results it is recommended that laboratories conform to international evidence based quality assurance (QA) guidelines on how tumor samples should be prepared, tested and reported for these biomarkers [1, 3, 4, 15]. One of the criterion stated in these guidelines is that laboratories carrying out these tests should participate in an external quality assessment (EQA) program [16], also known as performance testing (PT), such as the programs offered by the UK National External Quality Assessment scheme (UK NEQAS) [17], College of American Pathologists (CAP) [18] and the Royal College of Pathologists of Australasia (RCPA) [19]. In Vietnam, one of the main EQA programs offered to laboratories is run by the Center for Standardization and Quality Control in Medical Laboratory of Ho Chi Minh City (CSQL) [20]. However, prior to the current study, CSQL had not offered EQA for ER, PR, HER2 and Ki-67.

In order to assess the reliability of IHC testing for these important predictive and prognostic breast cancer biomarkers and in collaboration with the CSQL we organised a pilot EQA ring study involving hospitals from the south, north, and middle of Vietnam, estimated to be approximately half of all clinical hospitals in the country testing for these markers. Moreover, as cases are often referred for testing between hospitals where there are preparatory (pre-analysis) variables such as fixation and paraffin processing, we considered it imperative that the EQA study included the testing of samples prepared in all of the participating laboratories. The logistics of instigating this type of ring study, where each laboratory simultaneously tests not only its own cases but that of every other participating laboratory, is complex and not currently offered by established EQA programs. The hypotheses are that the assays are highly reproducible; by the same laboratory on the same cases when tested on separate occasions (intra-laboratory reproducibility) and by different laboratories testing the same cases (inter-laboratory reproducibility).

## Materials and Methods

### Participating Laboratories

The pathology departments from ten Vietnamese hospitals participated in this study; Ho Chi Minh City (HCMC) Oncology Hospital, Tu Du Hospital HCMC, Nhan dan Gia Dinh Hospital HCMC, Military Hospital 175 HCMC, Ca Mau General Hospital, Can Tho Oncology Hospital, Da Nang Oncology Hospital, Da Nang Hospital for Women and Children, Hue Central Hospital and 108 Military Central Hospital Ha Noi city.

### Tissue Samples

In June 2024 participating laboratories were requested to select from their files four surgically excised infiltrating ductal carcinomas (IDC) of the breast reported in the last 5 years and optimally fixed in neutral buffered formalin (NBF), or a similar formalin based fixative such as formal saline. It was recommended that fixation time should not be shorter than 8 h and no longer than 48 h [21]. Participants were instructed that selection of cases should be on the basis of the tumor expression of HER2 i.e., one case to have 3+ expression, one case 2+ (non-amplified), one case 1+ expression and one case 0 (zero) expression for HER2, respectively. Having selected the cases, participants were requested to produce a tissue micro array (TMA) of the 4 cases in order that, following microtomy, all 4 cases could be placed on one microscope slide. In addition to retesting the cases for HER2, the participants were requested to also re-test the cases for ER, PR and Ki-67. They were also asked to score the results for each marker using the recommended scoring guidelines and enter the data onto a form provided by the CSQL organizing center. Each laboratory then cut an additional 40 sections and mounted them onto appropriate microscope slides for IHC testing and returned them, along with the four stained slides and the completed forms, to CSQL.

### EQA Procedure

In July 2024 upon receiving the 4 stained and 40 unstained slides from each of the 10 participants, CSQL gave each participant a unique identifying number (UIN) in order to ensure anonymity during testing. The slides were then sorted by CSQL and 4 unstained slides from each of the 10 participants and comprising in total 40 different cases of invasive breast carcinoma were couriered to each of the 10 participating laboratories. Each participating laboratory then stained 10 different slides (40 cases) for HER2, 10 slides for ER, 10 slides for PR and 10 slides for Ki-67 using their usual protocol in addition to scoring the cases using the recommended scoring guidelines. Each participant then returned the 40 stained slides along with the scores and technical data to CSQL, by the specified deadline.

### Assessment

For ER, PR and HER2 testing, each laboratory scored the slides using guidelines recommended by the American Society of Clinical Oncology (ASCO) and CAP [2, 15]. For ER and PR this comprised the 8-point system, commonly referred to as the Allred score, which involves estimating the proportion of invasive tumor cells with nuclei staining, in addition to the intensity of tumor nuclear staining. Briefly, a score of 0 (zero) is given for no nuclear staining, 1 for <1%, 2 for 1%–10%, 3 for 11%–33%, 4 for 34%–66% and 5 for >66% of tumor nuclei staining. In addition, an intensity score of 0 (zero) is awarded for no nuclear staining, 1 for weak, 2 for moderate and 3 for strong nuclear staining of the tumor nuclei. The two scores are then added to give a maximum score of 8, and a minimum score of 0 (zero). Evidence from clinical studies have shown that patients with tumors having a score of ≥3 are likely to benefit from hormonal therapy [2, 22].

HER2 was assessed using the latest focus update guidelines for HER2 expression by IHC in invasive tumor cells, with a score of 3+ given for circumferential membrane staining that is complete, intense and in >10% of tumor cells, 2+ for weak to moderate complete membrane staining in >10% of tumor cells, 1+ for incomplete membrane staining that is faint/barely perceptible and in >10% of tumor cells, and 0 for no staining observed or membrane staining is incomplete and faint in 10% or less of tumor cells [1, 15].

Following discussion within the group of participating laboratories it was agreed that assessment of Ki-67 should be carried out by estimating the proportion of invasive tumor nuclei staining in the whole of the TMA section using intervals of 5%, ranging from 0% to 100%.

### Technical Analysis

The majority of participants used either the Ventana BenchMark GX (Roche Diagnostics, HCMC, Vietnam) or Ventana BenchMark Ultra (Roche Diagnostics) as the IHC automated staining platform, whilst two laboratories employed a manual method. Rabbit monoclonal antibodies were predominantly used; the SP1 clone for ER (Roche Diagnostics), the 1E2 clone for PR (Roche Diagnostics), the 4B5 clone (Roche Diagnostics) for HER2 and clone 30–9 for Ki67 (Roche Diagnostics). The Roche Ultra-View Universal DAB Kit was mainly used for detection, whilst Ventana Cell Conditioning 1 (CC1) Tris pH8 was the main antigen retrieval solution used for all four antibodies (Table 1).

**TABLE 1:** Technical details of the assays used by participating laboratories for the testing of estrogen receptors (ER), progesterone receptors (PR), human epidermal growth factor receptor-2 (HER2) and Ki-67 proliferating antigen at EQA. The number (n) of laboratories using each variable is shown.

	ER	PR	HER2	Ki-67
Automation	Ventana BenchMark GX (n = 3)	Ventana BenchMark GX (n = 3)	Ventana BenchMark GX (n = 3)	Ventana BenchMark GX (n = 3)
	Ventana BenchMark Ultra (n = 5)	Ventana BenchMark Ultra (n = 5)	Ventana BenchMark Ultra (n = 5)	Ventana BenchMark Ultra (n = 5)
	Manual (n = 2)	Manual (n = 2)	Manual (n = 2)	Manual (n = 2)
Antibody	Roche clone SP1 Rabbit monoclonal (n = 6)	Roche clone 1E2 Rabbit monoclonal (n = 8)	Roche clone 4B5 Rabbit monoclonal (n = 7)	Roche clone 30–9 Rabbit monoclonal (n = 6)
	BioSB clone BSB-1, mouse monoclonal (n = 1)	BioSB clone BSB-2, mouse monoclonal (n = 1)	BioSB clone BSB-2 mouse monoclonal (n = 1)	BioSB clone RM 360, Rabbit monoclonal(n = 1)
				BioSB clone EP5 Rabbit monoclonal(n = 1)
				Dako clone MIB1, Mouse monoclonal (n = 1)
	Full details not provided (n = 3)	Full details not provided (n = 1)	Full details not provided (n = 2)	Full details not provided (n = 1)
Detection	Roche UltraView Universal DAB Detection Kit (n = 6)	Roche UltraView Universal DAB Detection Kit (n = 6)	Roche UltraView Universal DAB Detection Kit (n = 6)	Roche UltraView Universal DAB Detection Kit (n = 6)
	Dako EnVision Flex High pH HRP DAB (n = 2)	Dako EnVision Flex High pH HRP DAB (n = 2)	Dako EnVision Flex High pH HRP DAB (n = 2)	Dako EnVision Flex High pH HRP DAB (n = 2)
	BioSB Mouse/Rabbit Poly Detector Plus DAB (n = 1)	BioSB Mouse/Rabbit Poly Detector Plus DAB (n = 1)	BioSB Mouse/Rabbit Poly Detector Plus DAB (n = 1)	BioSB Mouse/Rabbit Poly Detector Plus DAB (n = 1)
	Full details not provided (n = 1)	Full details not provided (n = 1)	Full details not provided (n = 1)	Full details not provided (n = 1)
Antigen retrieval	Ventana Cell Conditioning 1 (CC1) Tris pH8. (n = 6)	Ventana Cell Conditioning 1 (CC1) Tris pH8. (n = 6)	Ventana Cell Conditioning 1 (CC1) Tris pH8. (n = 6)	Ventana Cell Conditioning 1 (CC1) Tris pH8. (n = 6)
	Dako Target Retrieval Solution High pH (Tris EDTA pH9.0 (n = 1)	Dako Target Retrieval Solution High pH (Tris EDTA pH9.0 (n = 1)	Dako Target Retrieval Solution High pH (Tris EDTA pH9.0 (n = 1)	Dako Target Retrieval Solution High pH (Tris EDTA pH9.0 (n = 1)
	Full details not provided (n = 3)	Full details not provided (n = 3)	Full details not provided (n = 3)	Full details not provided (n = 3)

Key: Ventana/Roche Products, Roche Diagnostics, HCMC, Vietnam. BioSB, products, Technimex JSC, hanoi, Vietnam.

### Statistical Analysis

In line with other studies of agreement between pathologists in the scoring of IHC breast cancer biomarkers using Cohen’s kappa coefficient, Cohen’s interpretation of the results was used, as shown in Table 2 [23, 24]. The proportion (%) of cases for which scoring resulted in agreement was also recorded.

**TABLE 2:** Interpretation of Cohen’s kappa coefficient for inter-rater agreement.

Cohen’s kappa	Level of agreement
1.00	Perfect
0.81–0.99	Almost perfect
0.61–0.80	Substantial
0.41–0.60	Moderate
0.21–0.40	Fair
0.0–0.20	Slight

^a^ Data derived from reference 24.

### Intra-Laboratory Testing

In the testing of in-house tumors on two separate occasions, it was expected that each laboratory should achieve the exact same scores on both occasions. A weighted Cohen’s kappa coefficient was used to determine this level of agreement for the testing of HER2, ER, PR and Ki67.

### Inter-Laboratory Testing

The median value and interquartile range (IQR) were used to determine the distribution of the scores on the forty cases when tested by the ten participating laboratories (Table 3). The assessment of HER2 involves a scoring system with a relatively narrow range of values (0, 1+, 2+, 3+), all of which have clinical relevance [5, 15], therefore the median value was the expected result, with scores outside the median considered to be discordant (non-agreement). However, for the testing of hormonal receptors ER and PR with a wider range of values, not all of the scores have differing clinical relevance e.g., an Allred Score of 4, 5, 6, 7, or 8 are essentially equivalent in their reliability of predicting a favorable response to hormonal therapies [2, 22]. Therefore, agreement (concordance) for ER and PR was either a score equivalent to the median, or a score that fell in the same clinically relevant range as the median i.e., if in the testing of ER and PR the median score was ≥3, then the expected participants score was also a score ≥3 [22]^,^ this was then considered agreement. Alternatively, if the ER and PR median score was <3, then the expected participants score was also a score <3. The same rationale was used in the analysis of inter-laboratory data for the assessment of Ki-67, though using the clinically relevant cut-point of ≥30% for Ki67 expression, as evidenced by Probert et al (2023) [7] for prognosis and the clinically relevant cut-point of ≥20%, for predicting favorable response to abemaciclib combined with endocrine therapy, as evidenced by Johnston et al (2020) [25]. Cohen’s kappa coefficient was used to determine agreement. A P value of <0.05 for the results of the statistical tests, was considered to be significant. The mean, standard deviation and coefficient of variation were used to compare the proportions of cases for which the laboratories achieved the expected results.

**TABLE 3:** Distribution of results on forty cases of invasive breast cancer tested by ten Vietnamese laboratories for estrogen receptors (ER), progesterone receptors (PR), Human Epidermal Growth Factor Receptor −2 (HER2) and Ki-67 proliferating antigen.

Case	Type^a^	Median and inter-quartile range			
		ER	PR	HER2	Ki-67
1	HER2 enriched	0 [0–0]	0 [0–0]	3 [1.5–3]	40 [27.5–55]
2	TN/HER2 low	0 [0–0]	0 [0–0]	1 [0–2]	22.5 [17.5–40]
3	Luminal A/HER2 low	8 [6–8]	5 [3.5–5]	1 [0–1.5]	10 [5–20]
4	Luminal A	8 [6–8]	8 [8–8]	0 [0–0]	2 [0–7.5]
5	HER2 enriched	0 [0–0]	0 [0–0.5]	3 [2–3]	32.5 [20–60]
6	Luminal A/HER2 low	8 [7.75–8]	8 [7–8]	1 [0–1]	5 [4–10]
7	Luminal A	3 [0–3.5]	6 [6–7]	0 [0–1]	17.5 [8.75–30]
8	TN	0 [0–0]	0 [0–0]	0 [0–0]	65 [40–76.25]
9	HER2 enriched	6 [4–6.5]	3 [1.5–5]	3 [2–3]	20 [13.75–30]
10	Luminal A/HER2 low	4 [1–6]	3 [0–4]	1 [0–1.25]	5 [1–10]
11	Luminal A	7.5 [4.5–8]	8 [7–8]	0 [0–0.25]	10 [8.75–16.25]
12	Luminal B	7 [4.5–8]	0 [0–0]	0 [0–0]	3 [0.75–5.25]
13	HER2 enriched	0 [0–0]	0 [0–0]	3 [3–3]	55 [30–70]
14	Luminal A/HER2 low	7 [5.75–8]	6 [6–7.25]	1 [0–2]	35 [24.5–60]
15	Luminal A	8 [7–8]	7 [6.75–7]	0 [0–0.25]	5 [1.5–5]
16	TN	0 [0–0]	0 [0–0]	0 [0–0]	50 [23.75–90]
17	HER2 enriched	0 [0–5.25]	0 [0–0]	3 [3–3]	30 [10–30]
18	TN/HER2 low	0 [0–0]	0 [0–0]	1 [0–2]	45 [38.75–50]
19	Luminal A	8 [6.75–8]	6 [2.25–6.25]	0 [0–1]	20 [12.5–22.5]
20	Luminal A	7.5 [6–8]	4.5 [0–5]	0 [0–0]	50 [33.75–70]
21	TN/HER2 low	0 [0–3]	0 [0–3]	2 [2–3]	30 [11.25–45]
22	Luminal A	7.5 [6–8]	7 [6.75–8]	0 [0–0]	10 [2.5–17.5]
23	Luminal A	8 [6.75–8]	7 [6.75–8]	0 [0–0]	20 [7.5–27.5]
24	Luminal A	5 [0–7]	3 [0–4]	0 [0–0]	5 [0–7.5]
25	HER2 enriched	0 [0–0]	0 [0–0]	3 [3–3]	30 [18.75–31.25]
26	Luminal A/HER2 low	6.5 [5–7.25]	0 [0–0]	2 [1–2]	25 [18.75–31.25]
27	Luminal A/HER2 low	8 [6.75–8]	8 [6.75–8]	1 [1–2]	10 [5–25]
28	TN	0 [0–0]	0 [0–3.25]	0 [0–0]	75 [47.5–82.5]
29	HER2 enriched	0 [0–0]	0 [0–0]	3 [3–3]	10 [0.5–25]
30	Luminal A	7 [5.75–8]	7 [4.75–7]	0 [0–0]	5 [2–12.5]
31	TN	0 [0–0]	0 [0–0]	0 [0–0]	30 [6.25–55]
32	Luminal A	6 [3.75–6.25]	6 [4.5–7]	0 [0–0]	40 [23.25–42.5]
33	HER2 enriched	0 [0–0]	0 [0–0]	3 [3–3]	60 [50–72.5]
34	Luminal B/HER2 low	0 [0–0]	4 [0–5]	2 [1–2]	10 [10–20]
35	Luminal B/HER2 low	6 [4.75–7]	3 [0–4]	1 [1–1.25]	72.5 [60–82.5]
36	TN	0 [0–0]	3 (3-0–4.25]	0 [0–0.25]	70 [60–81.25]
37	HER2 enriched	0 [0–3.25]	3 [0–3]	3 [3–3]	40 [25–50]
38	TN/HER2 low	0 [0–3]	0 [0–0]	2 [2–2.25]	30 [5–62.5]
39	Luminal A/HER2 low	7 [6.75–8]	8 [7–8]	1 [0–1]	5 [3.75–11.25]
40	Luminal A	8 [6.75–8]	3 [1.5–3.25]	0 [0–0]	20 [13.75–30]

KEY.

^a^ Based on the results of biomarker testing. TN, triple negative.

## Results

**FIGURE 1:**
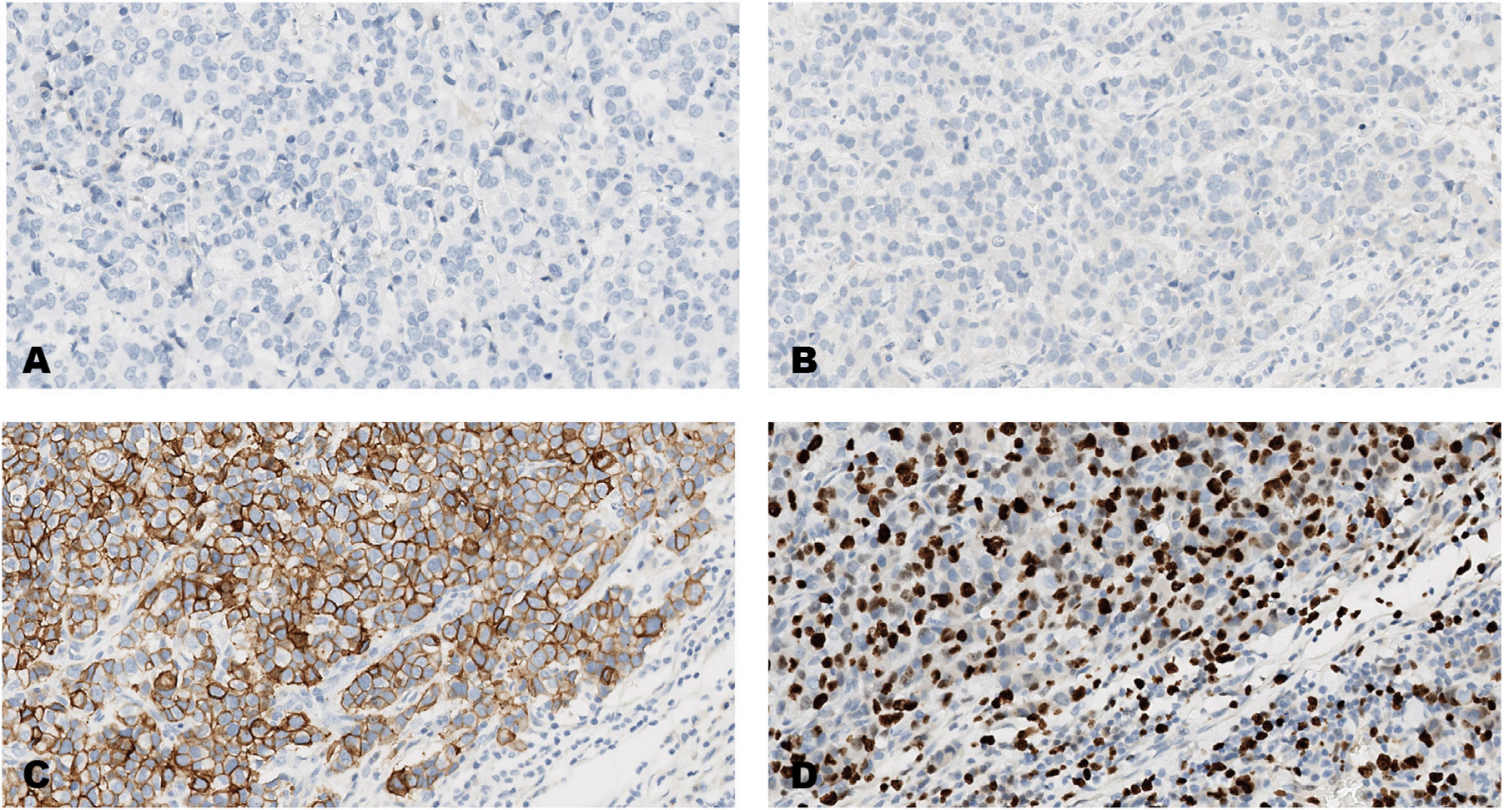
An invasive carcinoma of the breast tested for; **(A)** estrogen receptors, **(B)** progesterone receptors, **(C)** human epidermal growth factor receptor-2, **(D)** Ki-67 proliferating antigen. Following immunohistochemical testing and scoring by all ten participating laboratories, the median scores for the tumor were; (A, ER 0), (B PR 0), (C HER2 3+), (D Ki67 40%). Magnification ×20 (all images).

**FIGURE 2:**
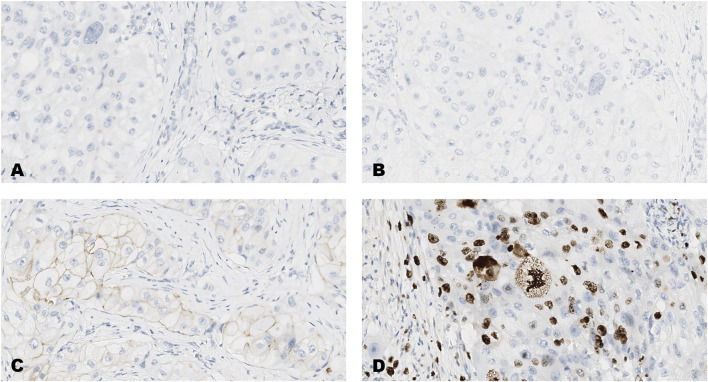
An invasive carcinoma of the breast tested for; **(A)** estrogen receptors, **(B)** progesterone receptors, **(C)** human epidermal growth factor receptor-2, **(D)** Ki-67 proliferating antigen. Following immunohistochemical testing and scoring by all ten participating laboratories, the median scores for the tumor were; (A, ER 0), (B PR 0), (C HER2 1+), (D Ki67 23%). Magnification ×20 (all images).

**FIGURE 3:**
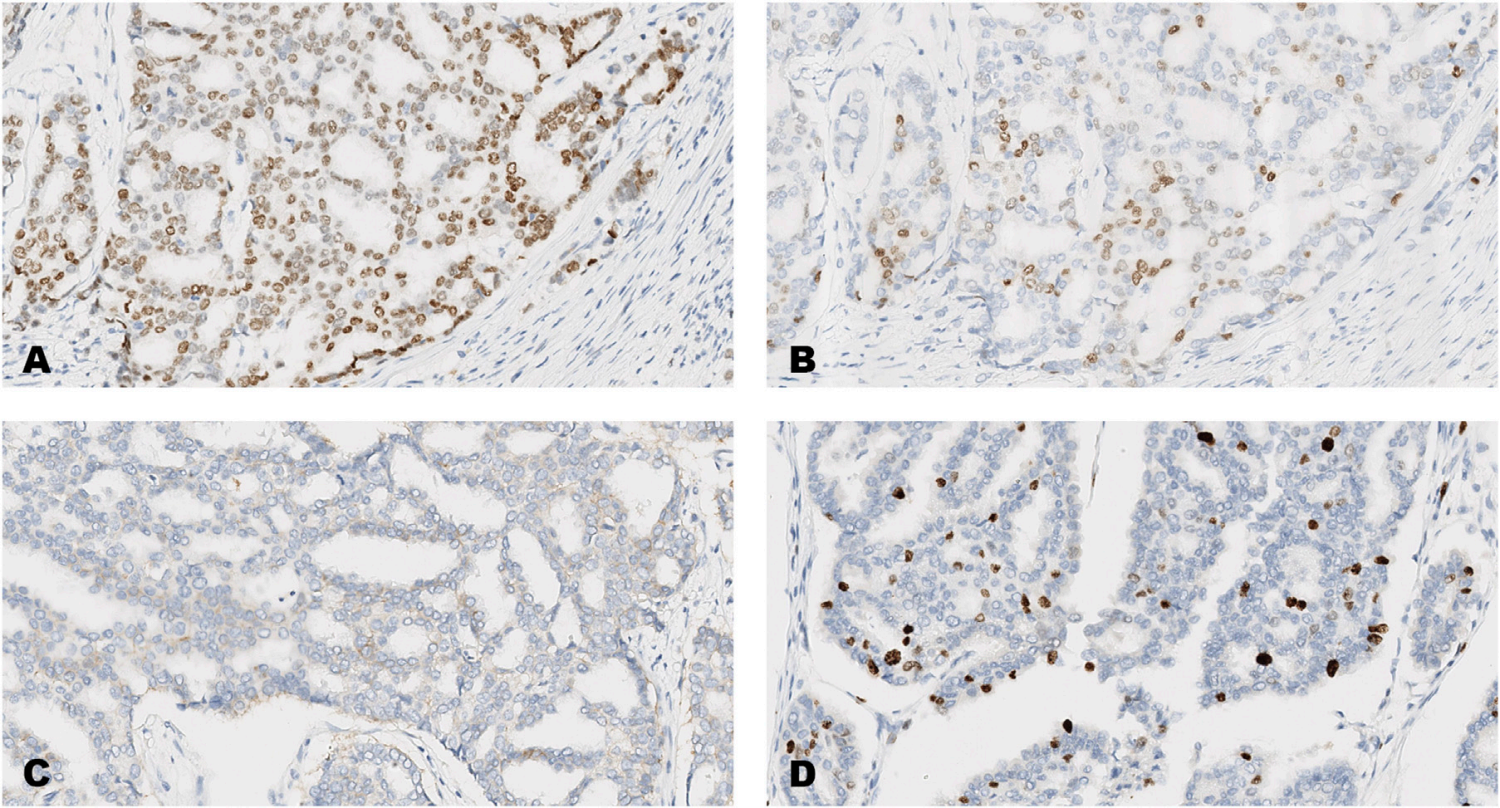
An invasive carcinoma of the breast tested for; **(A)** estrogen receptors, **(B)** progesterone receptors, **(C)** human epidermal growth factor receptor-2, **(D)** Ki-67 proliferating antigen. Following immunohistochemical testing and scoring by all ten participating laboratories, the median scores for the tumor were; (A, ER 8), (B PR 5), (C HER2 1+), (D Ki67 10%). Magnification ×20 (all images).

**FIGURE 4:**
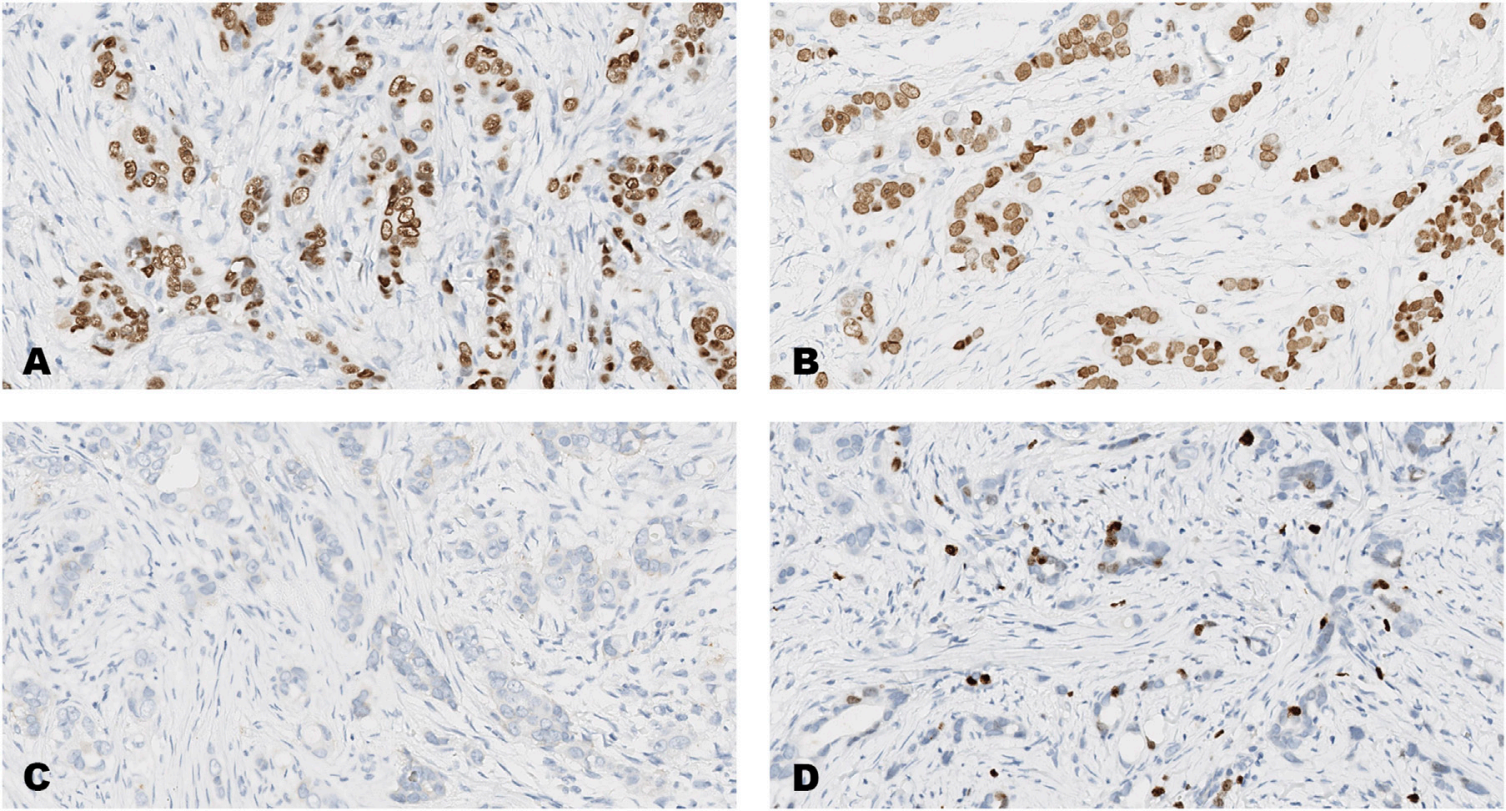
An invasive carcinoma of the breast tested for; **(A)** estrogen receptors, **(B)** progesterone receptors, **(C)** human epidermal growth factor receptor-2, **(D)** Ki-67 proliferating antigen. Following immunohistochemical testing and scoring by all ten participating laboratories, the median scores for each tumor were; (A ER 8), (B PR 8), (C HER2 0), (D Ki67 2%). Magnification ×20 (all images).

The distribution of scores on each of the forty-breast cancer cases for each of the four markers, ER, PR, HER2 and Ki-67 are shown in Table 3 and images of the results obtained on four of the forty tumors (case numbers 1-4, in Table 3) are shown in Figures 1–4. Based on the IHC profile (Table 3) the cases comprised; 19 luminal A, 3 luminal B, 9 HER2 enriched and 9 triple negative invasive breast carcinomas. Across, most profiles, there were 13 tumors with HER2 low expression (1+, or 2+ with no HER2 gene amplification).

### Intra-Laboratory Reproducibility of Testing

The reproducibility of testing of the forty different cases on two separate occasions (initial testing of the TMA *versus* the result achieved at the EQA run), was significant for all four antibodies. Greatest reproducibility was for HER2 testing (kappa 0.628), followed by PR (kappa 0.623), ER (kappa 0.488) and Ki-67 (0.215). Table 4.

**TABLE 4:** Intra-laboratory reproducibility of testing the same cases for; estrogen receptors (ER), progesterone receptors (PR), human epidermal growth factor receptor-2 (HER2) and Ki-67 proliferating antigen by the ten laboratories on cases of invasive breast cancer on two separate occasions.

Antibody	Complete agreement N (%)	Cohens weighted kappa	P
ER	23/38 (61)	0.448	<0.001
PR	29/40 (73)	0.623	<0.001
HER2	29/40 (73)	0.628	<0.001
Ki-67	13/36 (36)	0.215	<0.001

KEY: Number (N) of cases, Proportion (%) of cases for which there was complete agreement (identical scores).

### Inter-Laboratory Reproducibility of Testing

#### Hormonal Receptors

The inter-laboratory testing of ER and PR achieved a high level of overall agreement with 92% (kappa 0.822) agreement for ER and 88% (kappa 0.752) for PR. These markers also gave the lowest levels of variation of results between laboratories, with a CV of 4.8% for ER and a CV of 7.1% for PR (Table 5).

**TABLE 5:** Inter-laboratory reproducibility of hormonal receptor testing on forty cases of invasive breast carcinoma by each of the 10 participating laboratories, utilizing the Allred threshold score of ≥3.

Estrogen receptor agreement				Progesterone receptor agreement		
Laboratory	N (%)	Kappa*	P	N (%)	Kappa*	P
A	40/40 (100)	1	<0.001	34/40 (85)	0.688	<0.001
B	39/40 (98)	0.950	<0.001	34/38 (90)	0.792	<0.001
C	37/40 (93)	0.848	<0.001	37/40 (93)	0.848	<0.001
E	35/40 (88)	0.754	<0.001	32/40 (80)	0.614	<0.001
F	36/40 (90)	0.794	<0.001	39/40 (98)	0.948	<0.001
G	32/37 (87)	0.728	<0.001	35/39 (90)	0.791	<0.001
H	34/39 (87)	0.739	<0.001	35/40 (88)	0.754	<0.001
J	36/40 (90)	0.802	<0.001	35/40 (88)	0.750	<0.001
K	37/40 (93)	0.851	<0.001	30/40 (75)	0.525	<0.001
L	35/40 (88)	0.744	<0.001	36/39 (92)	0.836	<0.001
Overall	361/396 (92)	0.822	<0.001	347/396 (88)	0.752	<0.001
Mean % (SD)	91.4% (4.3)			87.9 (6.3)		
CV	4.8%			7.1%		

KEY: kappa coefficient, SD, standard deviation; CV, coefficient of variation. Number (N) and proportion (%) agreement.

#### HER2 Testing

Seventy-seven percent of the results were in agreement scores, with an overall kappa coefficient of 0.794 (95% CI 0.753–0.836), and a CV of 9.7% (Table 6).

**TABLE 6:** Inter-laboratory reproducibility of Human Epidermal Growth Factor Receptor-2 (HER2) testing on forty cases of invasive breast carcinoma by each of the 10 participating laboratories.

HER2 agreement				
Laboratory	N (%)	Kappa*	95% CI	P
A	27/40 (68)	0.795	0.688–.902	<0.001
B	33/40 (83)	0.861	0.764–.958	<0.001
C	32/40 (80)	0.848	0.751–.946	<0.001
E	30/40 (75)	0.787	0.658–.917	<0.001
F	30/36 (83)	0.826	0.676–.976	<0.001
G	24/40 (60)	0.703	0.576–.832	<0.001
H	34/40 (85)	0.839	0.734–.944	<0.001
J	30/40 (75)	0.699	0.530–.867	<0.001
K	32/39 (82)	0.816	0.680–.951	<0.001
L	32/40 (80)	0.776	0.614–.938	<0.001
Overall	304/395 (77)	0.794	0.753–0.836	<0.001
Mean % (SD)	77% (7.5)			
CV	9.7%			

KEY: weighted kappa coefficient, SD, standard deviation; CV, coefficient of variation. Number (N) and proportion (%) agreement.

### HER2 Low Cases

The scoring of the cases tested for HER2, showed there to be 13 cases with HER2 low expression and 27 cases for which the scores were either 3+ or 0 (Table 3). Excluding 5 technical failures, this resulted in 127 and 268 individual pathologist assessments for HER2 low and HER2 3+ and 0 cases, respectively. Just 50% of the HER2 low scores were in agreement, compared to 89% of the HER2 3+ and 0 scores. Using Cohen’s kappa statistic, the level of agreement for the HER2 low cases (1+, 2+) was kappa 0.323 (95% CI 0.223–0.424) compared to kappa 0.879 (95% CI 0.835–0.923) for the scoring of HER2 3+ and 0 cases (Table 7).

**TABLE 7:** Inter-laboratory reproducibility of scoring Human Epidermal Growth Factor Receptor-2 low (HER2 low) and HER2 3+ and 0 cases on cases of invasive breast carcinoma by all 10 participating laboratories.

HER2 low (1+, 2+)				HER2 3+ and 0			
N (%)	Kappa*	95% CI	P	N (%)	Kappa*	95% CI	P
64/127 (50)	0.323	0.223–.424	<0.001	239/268 (89)	0.879	0.835–.923	<0.001

KEY: weighted kappa coefficient, Number of assessments (N) and proportion (%) of assessments in agreement, Confidence Interval (CI).

#### Ki-67 Testing

Eighty-four percent of the results were in agreement, with an overall kappa coefficient of 0.647 and a CV of 17%, when using the cut point of ≥30% to define a positive result. In comparison, 81% of the results were in agreement, with an overall kappa coefficient of 0.601 and a CV of 12.1% when using the cut point of ≥20% to define a positive result (Table 8).

**TABLE 8:** Inter-laboratory reproducibility of Ki-67 proliferating antigen testing on forty cases of invasive breast carcinoma by each of the 10 participating laboratories, utilizing the thresholds of ≥30% and ≥20% for positivity of invasive tumor cells.

Ki67 agreement (≥30% threshold)				Ki67 agreement (≥20% threshold)		
Laboratory	N (%)	Kappa*	P	N (%)	Kappa*	P
A	31/40 (78)	0.509	<0.001	30/40 (75)	0.403	0.004
B	30/38 (79)	0.578	<0.001	30/38 (79)	0.559	0.001
C	31/40 (78)	0.496	0.002	31/40 (78)	0.550	<0.001
E	31/39 (80)	0.540	0.001	30/39 (77)	0.474	0.003
F	36/40 (90)	0.798	<0.001	35/40 (88)	0.744	<0.001
G	38/40 (95)	0.851	<0.001	38/40 (95)	0.893	<0.001
H	30/36 (83)	0.670	<0.001	26/36 (72)	0.456	0.002
J	35/40 (88)	0.746	<0.001	35/40 (88)	0.750	<0.001
K	38/39 (97)	0.897	<0.001	37/39 (95)	0.885	<0.001
L	28/40 (70)	0.386	0.007	25/40 (63)	0.318	0.013
Overall	328/392 (84)	0.647	< 0.001	317/392 (81%)	0.601	<0.001
Mean % (SD)	81.3% (13.8)			81.0% (9.8)	81.0%	
CV	17.0%			12.1%		

KEY: Cohen’s kappa coefficient, SD, standard deviation; CV, coefficient of variation. Number (N) and proportion (%) of scores in agreement.

## Discussion

In the current study the CSQL organized an EQA ring study to determine the reliability of testing for ER, PR, HER2 and Ki-67 in ten hospitals from the south, middle and north of Vietnam, estimated to represent approximately half of all Vietnamese hospital laboratories currently testing clinical cases for ER, PR, HER2 and Ki-67. As cases are often referred between hospital laboratories for testing we tested the reliability of testing on tissues prepared in all ten of the participating laboratories comprising four different cases from each laboratory (forty tumors in total). Consequently, a more rigorous assessment than the testing of single slides, comprising smaller numbers of tissues and often prepared in just one laboratory, as offered by many EQA programs.

McHugh (2012) [26], recommends using both proportional (percentage) agreement and Cohen’s kappa coefficient for determining the level of inter-rater agreement, as both have their strengths and limitations, and we followed this recommendation in the current study (Tables 4–8).

Whilst the reproducibility of testing of the same forty different cases on two separate occasions (intra-laboratory reproducibility), was significant for all four antibodies, it was by no means identical, as shown by the relatively low kappa scores, with greatest variation seen with the testing of Ki67.

The inter-laboratory reproducibility of testing, using clinically relevant cut-points for defining likely benefit to treatment strategies, was relatively high. Almost perfect agreement (kappa score of 0.81–1.00) was achieved for the testing and scoring of ER (kappa 0.822), with 92% (CV 4.8%), of results in agreement. Only one laboratory (laboratory A in Table 5) achieved the expected result on all forty cases of breast cancer, and this for the testing of ER. Similar to the intra-laboratory results, greatest variation was seen with Ki-67 testing (Table 8).

For over twenty-years HER2 has been assessed in clinical laboratories most commonly using a combined IHC and *in situ* hybridization (ISH) approach that classifies invasive breast carcinomas as either HER2 positive (3+ and 2+ cases with HER2 gene amplification) or HER2 negative (0, 1+ and 2+ cases without HER2 gene amplification) [1, 3]^.^ More recently, the results of the DESTINY-Breast 04 clinical trial have shown significant improvement in progression free survival and overall survival for women with advanced and metastatic breast cancer with tumors expressing low levels of HER2 expression (1+ and 2+ without HER2 gene amplification) and treated with the antibody drug conjugate, trastuzumab deruxtecan (T-DXd), compared to a control group receiving the physicians choice of therapy [5]. Subsequently, T-DXd has been approved by both the US Food and Drug Administration (FDA), the European Medicines Agency and the UK National Institute for Health and Care Excellence (NICE) for patients with unresectable or metastatic breast cancer [27, 28]. Some of the latest pathology guidelines for reporting HER2 from both the US and the UK emphasize the importance of appropriately scoring HER2 low breast cancers [15, 29]. Consequently, for HER2, each of the four scores; 0, 1+, 2+, 3+ now have clinical implications and therefore each is of importance. Therefore, for the assessment of HER2 in the current EQA the expected result was the central value only, the median, which in our study was also equivalent to the mode i.e., the single discreet score achieved by the majority of participants.

Overall, 77% of pathologists scores for HER2 were in agreement (Table 6), with a kappa concordance value of 0.794 (95% CI 0.753–0.836); a level of agreement considered as substantial by many studies utilizing Cohen’s kappa coefficient to compare agreement amongst pathologists. However, the level of agreement in the scoring of just the HER2 low cases was significantly lower, with only 50% of scores in agreement and with a kappa value of 0.323 (95% CI 0.223–0.424). In comparison, the scoring of HER2 3+ and HER2 0 was almost perfect (kappa 0.879, 95% CI 0.835–0.923), with 89% of scores in agreement (Table 7). Consensus agreement amongst a group of expert UK pathologists scoring HER2 have reported similar findings [23]. These results question the reliability of the IHC assay to accurately predict benefit to T-DXd for breast cancer patients. A conclusion similar to that reached by other authors [30, 31].

The International Ki67 in Breast Cancer Working Group (IKWG) suggest that the use of Ki-67 IHC, as opposed to more expensive multi-gene assays, is adequate for determining prognosis for breast cancer patients with ER positive disease with very low (5%) or very high (≥30%) Ki-67 scores. However, they recommend that inter-laboratory variation of the IHC assays is too great to rely on intermediate Ki-67 scores falling between these two values [6]. Indeed, in the current study testing for Ki-67 resulted in greater variation, than in the testing for HER2, ER and PR (Tables 5, 6, 8). Technical differences in the assays employed by the ten participating laboratories may have accounted for some of this variation, most noticeably the use of at least four different Ki-67 antibody clones, compared to the use of just two different clones for the other markers (Table 1). Evidence suggests that some of the Ki-67 antibody clones commercially available are more reliable than others [32]. Difference in antigen retrieval stringency may also have played a role, as an appropriate antigen retrieval step is essential for this marker [6, 33]. The importance of choosing the most appropriate antigen retrieval protocol for ER, PR and HER2 is also well documented [11, 34] and those laboratories that failed to achieve the expected result have been recommended to check to ensure that their assays are fully optimized, particularly with respect to choice of antibody and antigen retrieval protocol.

In Vietnam the testing and scoring of Ki-67 is not as established as for the other three markers. No doubt this also added to the increased variation in results. In this respect, whilst the authors of this paper are familiar with the latest IKWG guidelines, and similar initiatives to standardise Ki-67 scoring, they have found the 5% interval score that makes a global estimation, without counting specific cells, to be the most practical and convenient method by which to record Ki-67 expression [6, 35]. Indeed, there is some evidence to show that histopathologists can accurately discriminate between proportions that are only 5% different, albeit in equal sized images [36].

Recently Probert et al (2023) [7] investigated Ki-67 expression and breast cancer mortality in over 8,000 United Kingdom National Health Service (NHS) patients with ER positive and HER2 negative disease. This study found that there was very little difference in adjusted cumulative breast cancer mortality risk for women with 0%–5%, 6%–10%, 11%–19% and 20%–29% Ki67 positive tumors. In comparison, patients having tumors with higher Ki-67 scores of 30%–39% and 40%–100% had significantly greater mortality risk. Interestingly, standardization to guard against inter-laboratory variability had little impact on the results [7].

In our EQA ring study, whilst Ki-67 testing resulted in a greater level of variation in results than for the other three markers, employing the clinically relevant cut point of ≥30% for prognosis as used by Probert et al [7] resulted in an overall agreement of 84% for testing and scoring of the tumors. This is an important finding as whilst there is still room for considerable improvement in consistency of Ki-67 testing by Vietnamese laboratories, it does support the findings made by Probert et al (2023) [7] i.e., that interlaboratory variation may be less of a problem than previously thought if a simple cut point for prognosis is used and reinforces the potential use of Ki-67 on a more regular basis for clinical decision making. When using the cut point of ≥20% of Ki67 stained cells, as used to predict favorable response to abemaciclib combined with endocrine therapy for the adjuvant treatment of hormone positive, HER2 negative, node-positive, high-risk, early breast cancer [25], the level of overall agreement in our EQA ring study was lower, though still relatively high with an overall agreement of 81% for Ki-67 testing.

These are important findings for developing countries such as Vietnam, as IHC markers are considerably less expensive than multi-gene assays, such as Oncotype Dx [35]. In a population of over 90 million people, with over 24,000 new cases of breast cancer each year and rising [37], and with the financial burden of cancer care frequently falling on the shoulders of the patient [38], estimating Ki-67 by IHC would be a far more affordable way to determine prognosis and predict response to therapy than the use of expensive multi-gene assays. Going forward, it also emphasises the importance of EQA initiatives to help ensure the reliability of Ki-67 testing.

In summary, this is the first EQA ring study held within Vietnam for ER, PR, HER2 and Ki67 and sets the base line as to the current level of reproducibility for these assays within the country. The study reports on the level of both intra- and inter-laboratory reproducibility and utilised tissue samples prepared in all of the participating laboratories. There was a significant level of reproducibility for all four biomarkers, with ER testing giving the least variation and Ki67 the greatest variation in results. However, the reproducibility of testing cases for HER2-low expression was relatively poor. As there is evidence that participation in EQA improves the quality of results [11], we are confident that continued participation in the program will increase the level of reproducibility for these important biomarkers and help ensure their reliability for current and future clinical use. This work represents an advance in biomedical science because it provides evidence for the reproducibility of breast cancer biomarker testing in Vietnam.

## Summary Table

### What Is Known About This Subject

- Testing for ER, PR, HER2 and Ki67 on breast cancer samples is carried out in the majority of laboratories to predict response to therapies and for prognosis
- Issues surrounding the reproducibility of testing are well documented
- Guidelines recommend participation in EQA in order to ensure reliability of results

### What This Paper Adds

- The first EQA ring study for these biomarkers held within Vietnam
- Significant reproducibility for all 4 biomarkers
- When using clinically relevant cut points, over 80% of the Ki-67 evaluations are in agreement
- Reproducibility of testing for HER2 low expression is poor compared to overall agreement for HER2 testing

## Data Availability

The raw data supporting the conclusions of this article will be made available by the authors, without undue reservation.
