# Corrigendum: Reproducibility of Immunohistochemical Testing of Estrogen Receptors, Progesterone Receptors, Human Epidermal Growth Factor Receptor-2 (HER2) and Ki-67 in Vietnam

**DOI:** 10.3389/bjbs.2025.15864

**Published:** 2025-12-02

**Authors:** Thai Anh Tu, Nguyen Van Tin, Anthony Rhodes, Dinh Bui Quynh Anh, Le Thi Hong Dao, Nguyen Thi Truc Linh, Dinh Thi Khanh Nhu, Nguyen Thi Hong Nhung, Lam Thanh Cam, Ngo Thi Minh Hanh, Pham Nguyen Cuong, Nguyen Thanh Toan, Nguyen Khac Tuyen, Do Dinh Khanh, Tran Thi Truc Ngan, Lam Kieu Mong Thy, Nguyen Van Thanh, Nguyen Quang Tuan, Vo Ngoc Nguyen, Le Thi Thuy Nhu, Nguyen Dam Chau Bao

**Affiliations:** 1 Department of Pathology, Ho Chi Minh City (HCMC) Oncology Hospital, Ho Chi Minh City, Vietnam; 2 Bureau of Accreditation Vietnam, Center for Standardization and Quality Control in Medical Laboratory of Ho Chi Minh City (CSQL), Ho Chi Minh City, Vietnam; 3 Institute of Biomedical Sciences, London, United Kingdom; 4 Department of Pathology, Ca Mau General Hospital, Ca Mau, Vietnam; 5 Department of Pathology, 108 Military Central Hospital, Ha Noi, Vietnam; 6 Department of Pathology, Hue Central Hospital, Hue, Vietnam; 7 Department of Pathology, Military Hospital 175, Ho Chi Minh City, Vietnam; 8 Department of Pathology, Nhan Dan Gia Dinh Hospital, Ho Chi Minh City, Vietnam; 9 Department of Pathology, Can Tho Oncology Hospital, Can Tho, Vietnam; 10 Department of Pathology, Tu Du Hospital, Ho Chi Minh City, Vietnam; 11 Department of Pathology, Da Nang Oncology Hospital, Da Nang, Vietnam; 12 Department of Pathology, Da Nang Hospital for Women and Children, Da Nang, Vietnam

**Keywords:** ER, PR, HER2, Ki67, breast cancer

In the published article, there was an error in the legends for Figures 1–4, as published. The legends incorrectly refer to “Four invasive ductal carcinomas,” each figure should instead refer to just one invasive carcinoma. The corrected legends appear below.

**FIGURE 1:**
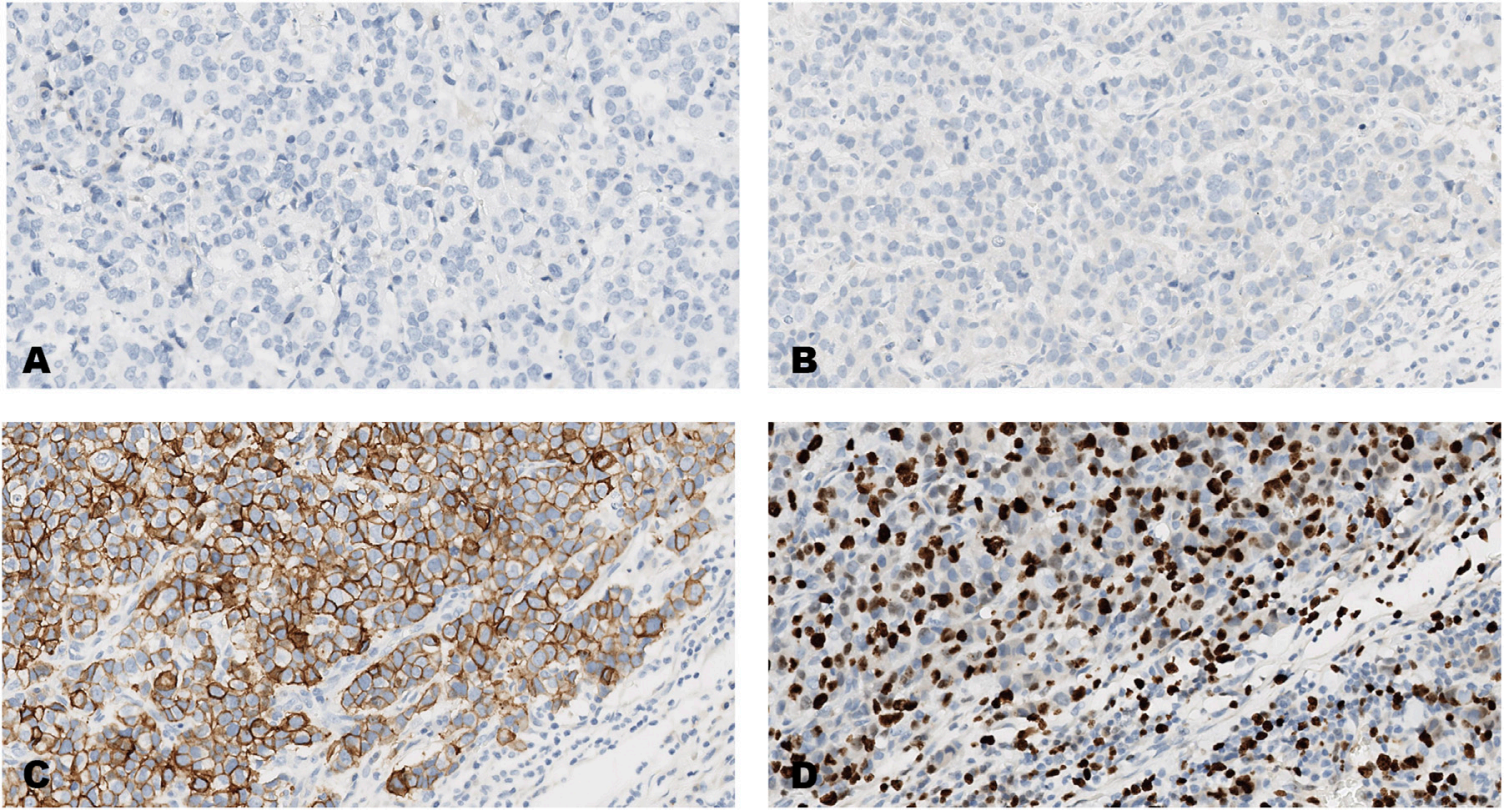
An invasive carcinoma of the breast tested for; **(A)** estrogen receptors, **(B)** progesterone receptors, **(C)** human epidermal growth factor receptor-2, **(D)** Ki-67 proliferating antigen. Following immunohistochemical testing and scoring by all ten participating laboratories, the median scores for the tumor were; (A, ER 0), (B PR 0), (C HER2 3+), (D Ki67 40%). Magnification ×20 (all images).

**FIGURE 2:**
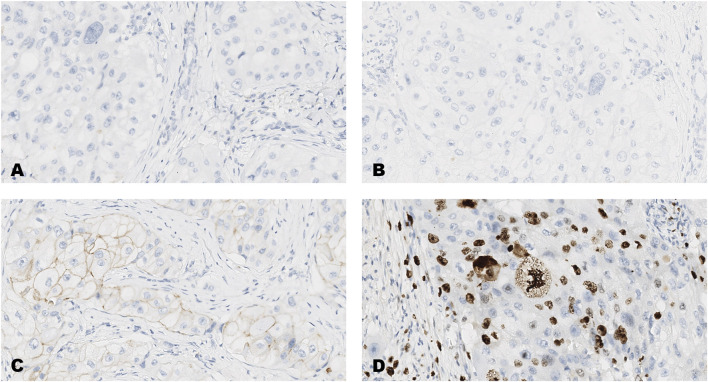
An invasive carcinoma of the breast tested for; **(A)** estrogen receptors, **(B)** progesterone receptors, **(C)** human epidermal growth factor receptor-2, **(D)** Ki-67 proliferating antigen. Following immunohistochemical testing and scoring by all ten participating laboratories, the median scores for the tumor were; (A, ER 0), (B PR 0), (C HER2 1+), (D Ki67 23%). Magnification ×20 (all images).

**FIGURE 3:**
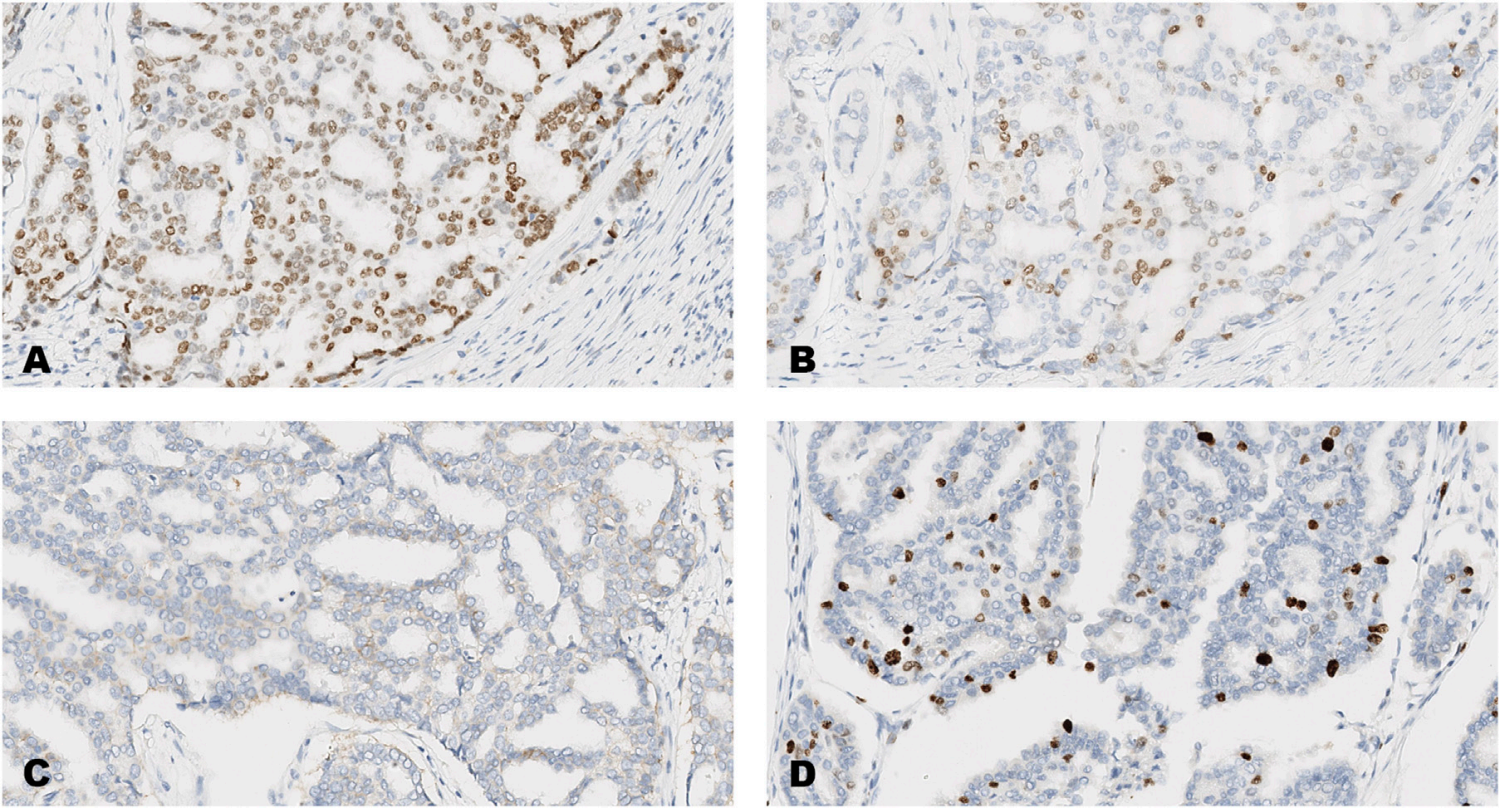
An invasive carcinoma of the breast tested for; **(A)** estrogen receptors, **(B)** progesterone receptors, **(C)** human epidermal growth factor receptor-2, **(D)** Ki-67 proliferating antigen. Following immunohistochemical testing and scoring by all ten participating laboratories, the median scores for the tumor were; (A, ER 8), (B PR 5), (C HER2 1+), (D Ki67 10%). Magnification ×20 (all images).

**FIGURE 4:**
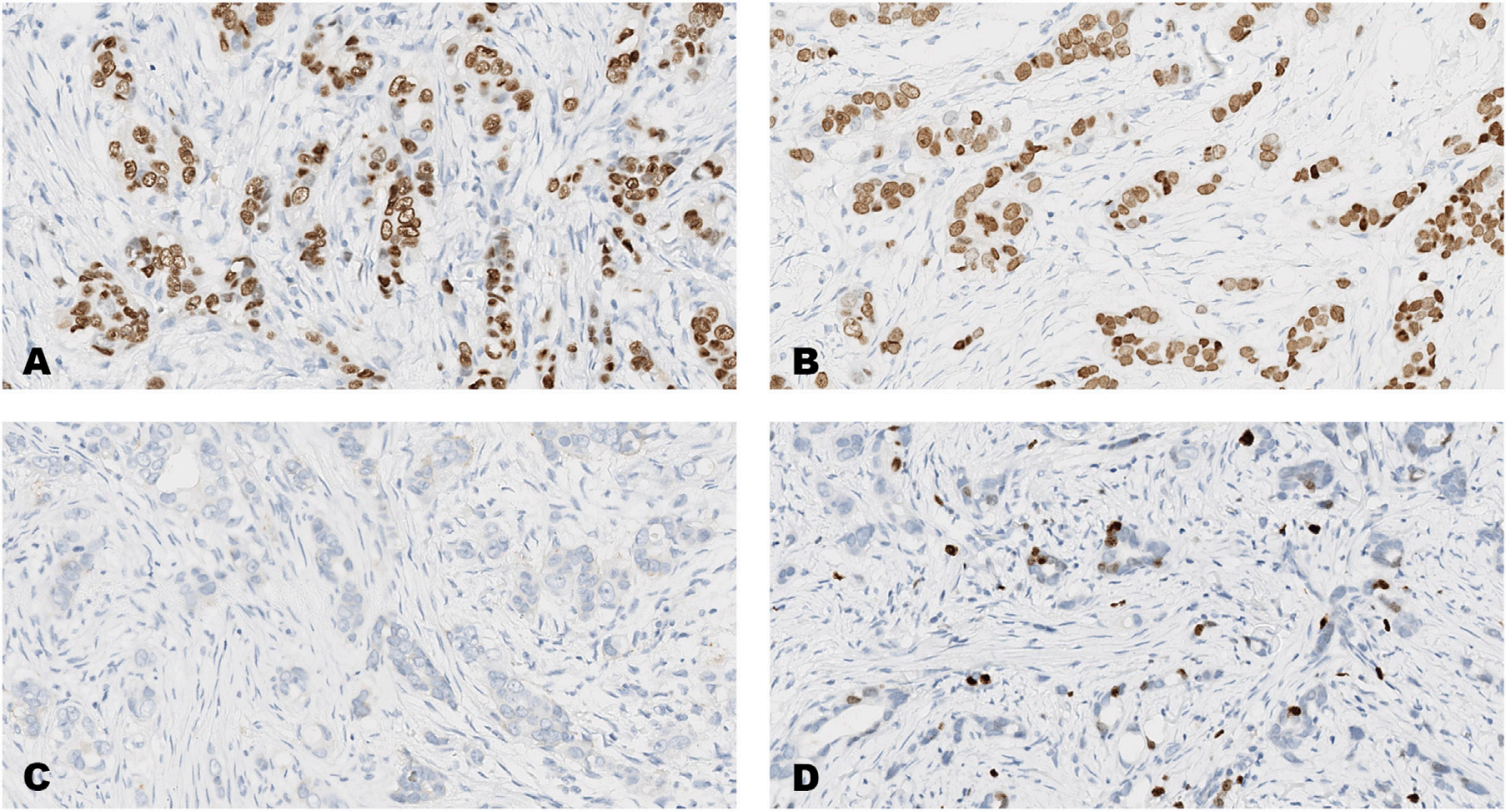
An invasive carcinoma of the breast tested for; **(A)** estrogen receptors, **(B)** progesterone receptors, **(C)** human epidermal growth factor receptor-2, **(D)** Ki-67 proliferating antigen. Following immunohistochemical testing and scoring by all ten participating laboratories, the median scores for each tumor were; (A ER 8), (B PR 8), (C HER2 0), (D Ki67 2%). Magnification ×20 (all images).

In the published article, the reference for Do, Whittaker and David, 2024 was incorrectly listed as reference number 37, and the reference for GLOBOCAN, 2022 was incorrectly listed as reference number 38. These should be reversed.

A correction has been made to **Materials and Methods**, Tissue Samples. At the end of the sentence “It was recommended that fixation time should not be shorter than 8 h and no longer than 48 h”, reference 22 was erroneously cited, instead reference 21 should be cited.

A correction has also been made to section **Materials and Methods**, *Inter-Laboratory Testing*. At the end of the sentence “Allred Score of 4, 5, 6, 7, or 8 are essentially equivalent in their reliability of predicting a favorable response to hormonal therapies” reference 21 was erroneously cited, instead reference 22 should be cited.

The authors apologize for these error and state that this does not change the scientific conclusions of the article in any way. The original article has been updated.

## Generative AI Statement

Any alternative text (alt text) provided alongside figures in this article has been generated by Frontiers with the support of artificial intelligence and reasonable efforts have been made to ensure accuracy, including review by the authors wherever possible. If you identify any issues, please contact us.

